# Heart Rate Variability as a Predictor of Region-Specific Brain Injury in Neonates with Perinatal Asphyxia: A Prospective Study in a Middle-Income Country

**DOI:** 10.3390/medicina61091631

**Published:** 2025-09-09

**Authors:** Sergio Agudelo-Pérez, Gloria Troncoso, Alvaro Arenas Auli, Camila Ayala

**Affiliations:** 1 Department of Pediatrics, School of Medicine, Universidad de La Sabana, Chía 250001, Cundianamarca, Colombia; 2 Head Neonatal Unit, Fundación Cardioinfantil—Instituto de Cardiología, Bogotá 110131, Colombia; gtroncoso@lacardio.org; 3 Pediatric Electrophysiology, Fundación Cardioinfantil—Instituto de Cardiología, Bogotá 110131, Colombia; aarenas@lacardio.org; 4 School of Medicine, Universidad de La Sabana, Chía 250001, Cundianamarca, Colombia; camilaayago@unisabana.edu.co

**Keywords:** perinatal asphyxia, hypoxic–ischemic encephalopathy, heart rate variability, middle-income countries, therapeutic hypothermia, magnetic resonance imaging

## Abstract

*Background and Objectives*: Neonates with moderate-to-severe hypoxic–ischemic encephalopathy (HIE) in low- and middle-income countries (LMICs) remain at high risk of neurological sequelae despite access to therapeutic hypothermia (TH). Real-time accessible biomarkers are required to improve risk stratification and guide neuroprotective care in these settings. This study evaluated the predictive capacity of heart rate variability (HRV) metrics for brain injury detected using magnetic resonance imaging (MRI) in neonates with HIE who underwent TH at an LMIC. *Materials and Methods*: We conducted a prospective observational study of 87 neonates treated with TH in a tertiary neonatal intensive care unit in Colombia. HRV was recorded during the first 24 h of TH, during rewarming, and 24 h after rewarming. Brain MRI was performed within the first week of life and scored using the Rutherford system. Associations between HRV metrics and global and regional brain injuries were analyzed using receiver operating characteristic (ROC) curves and multivariable logistic regression models. *Results*: Low-frequency (LF) and high-frequency (HF) powers were significantly lower in neonates with MRI abnormalities. LF power during rewarming demonstrated the highest predictive accuracy (AUC = 0.90), followed by HF power during the first 24 h (AUC = 0.80). Region-specific analyses showed that LF power reduction was significantly associated with white matter and basal ganglia injury. *Conclusions*: HRV, particularly LF power during rewarming, is a promising and accessible biomarker for regional brain injury in neonates with perinatal asphyxia treated with TH.

## 1. Introduction

Perinatal asphyxia remains a leading cause of neonatal mortality, accounting for an estimated 1.2 million intrapartum-related deaths annually, most of which occur in low- and middle-income countries (LMICs), with a prevalence ranging from 15.9% to 35% [1,2,3]. Survivors in these settings face a disproportionately high risk of medium- and long-term neurological sequelae despite access to therapeutic interventions [4,5]. 

Therapeutic hypothermia (TH) is the standard of care for neonates with moderate-to-severe perinatal asphyxia and hypoxic–ischemic encephalopathy (HIE) [6]. However, its effectiveness in LMICs is limited, likely because of variations in implementation, infrastructure, and quality of neonatal care [7,8]. These challenges are particularly relevant in settings in which many deliveries occur in first- or second-level hospitals with limited resources.

Therefore, early prediction of brain injury and timely risk stratification are critical for enabling individualized neuroprotective strategies, especially in high-burden settings with suboptimal responses to TH [9,10,11]. Although several biomarkers, such as magnetic resonance imaging (MRI), electroencephalography (EEG), and serum markers, have shown prognostic value [12,13,14,15], their clinical utility is often constrained by their high cost, technical requirements, and variable accuracy [16,17]. Thus, accessible, real-time bedside tools are urgently required for timely risk stratification during the early postnatal period [18,19,20].

Heart rate variability (HRV), which reflects the complex interplay between the sympathetic and parasympathetic nervous systems, has emerged as a promising, non-invasive, and low-cost biomarker for monitoring neurological function in neonates with hypoxic–ischemic encephalopathy (HIE) [21]. Specifically, HRV analysis during therapeutic hypothermia (TH) has been established as a real-time bedside tool for the early risk stratification of neurological injury [21,22].

However, research on HRV in the context of HIE has significant limitations. A key challenge is the scarcity of studies focusing on the predictive capacity of HRV for detecting specific regional brain injuries such as those affecting the basal ganglia or white matter, which are closely associated with adverse long-term neurological outcomes. Furthermore, most existing evidence originates from high-income settings, and its applicability in low- and middle-income countries (LMICs) remains unclear, underscoring the need to validate HRV as a context-appropriate prognostic marker [21,22,23].

Although HRV monitoring is a relatively straightforward method, the implementation and standardization of signal acquisition and analysis protocols in LMICs face unique challenges, including equipment availability and staff training. The paucity of research in these environments prevents a clear understanding of whether HRV metrics influenced by factors, such as therapeutic hypothermia, can consistently serve as predictors of regional brain injury. This study sought to address this gap by evaluating the predictive value of HRV parameters for specific regional brain injuries, quantified through cerebral magnetic resonance imaging and the Rutherford scoring system, in neonates with HIE in a middle-income country.

The objectives of this study were: (1) to evaluate the predictive capacity of HRV during TH to detect brain abnormalities on MRI in the first week of life in neonates with moderate-to-severe perinatal asphyxia, and (2) to examine the association between HRV parameters and MRI findings in this population. We hypothesized that specific HRV metrics, particularly low-frequency (LF) power, high-frequency (HF) power, and the LF/HF ratio, are associated with MRI-detected abnormalities.

## 2. Materials and Methods

### 2.1. Study Design and Setting

We conducted a prospective observational study between May 2022 and December 2023 in a level III neonatal intensive care unit (NICU) in Bogotá, Colombia, a tertiary referral center in a middle-income country. This study adhered to international therapeutic hypothermia (TH) protocols for neonatal encephalopathy and included access to magnetic resonance imaging (MRI). Ethical approval was obtained from the institutional ethics committee (CEIC-0195-2022) and written informed consent was obtained from the parents.

### 2.2. Participants

Eligible neonates were term or late preterm neonates (≥35 weeks, Ballard score) with moderate to severe perinatal asphyxia. Inclusion criteria were as follows: (a) umbilical pH ≤ 7.0, base deficit ≤ −16, postnatal pH 7.01–7.15 and/or base deficit −10 to −15.9, (b) sentinel perinatal event, (c) Apgar score ≤ 5 at 10 min, and (d) hypoxic–ischemic encephalopathy stage II–III according to the Sarnat criteria. The exclusion criteria were birth weight < 1800 g, major anomalies, CNS malformations, heart defects, or chromosomal disorders.

### 2.3. Procedures

Continuous heart rate monitoring was performed using a Holter ECG device (Cardioscan Premier II, DM Software Inc., Nevada, USA) during three predefined phases of treatment: (1) within the first 24 h of TH, (2) during rewarming, and (3) within 24 h after rewarming. For each phase, we acquired one contiguous 6 h ECG epoch, which was predefined as the representative analytic window for that phase (i.e., segments were not stitched from shorter intervals).

Signals were processed in MATLAB (MathWorks Inc., Natick, MA, USA) (R-peak detection, digital filtering for artifact correction, and baseline removal) and exported to Kubios HRV Scientific Lite 3.6 (Kubios Oy, Kuopio, Finland) for analysis.

HRV parameters were computed in both time and frequency domains. Time-domain metrics included the standard deviation of NN intervals (SDNN), root mean square of successive differences (rMSSD), and the percentage of NN intervals differing by >50 ms (pNN50). The frequency-domain metrics included low-frequency (LF: 0.04–0.15 Hz), high-frequency (HF: 0.15–0.40 Hz), and the LF/HF ratio.

### 2.4. Outcome Measures

The primary outcome was brain injury identified on MRI performed within the first week of life. Scans were acquired using the Philips Achieva dStream system and included both structural and advanced sequences. The structural sequences were T1-weighted, T2-weighted (turbo spin-echo), and Fluid-Attenuated Inversion Recovery (FLAIR). Advanced sequences included diffusion-weighted imaging (DWI), apparent diffusion coefficient (ADC), and Gradient-Echo T2 sequences. All images were obtained in the sagittal, axial, and coronal planes for comprehensive evaluation.

Brain injury was classified using the Rutherford scoring system, which evaluates four regions: the basal ganglia/thalamus (BG/T), white matter, cortex, and posterior limb of the internal capsule (PLIC), each scored on a 0–3 scale. For the analysis, the scores were dichotomized as 0 = no injury and 1 = any injury (scores 1–3). Clinical and demographic data (birth weight, gestational age, Apgar scores, and perinatal events) were extracted from electronic medical records.

### 2.5. Statistical Analysis

Descriptive statistics were used to summarize baseline characteristics. Categorical variables were reported as counts and percentages and continuous variables as means ± standard deviations or medians with interquartile ranges, as appropriate. Normality was assessed using the Shapiro–Wilk test.

Baseline characteristics were compared according to global MRI findings (normal vs. abnormal) using Student’s *t*-test or Mann–Whitney U test for continuous variables, and χ^2^ or Fisher’s exact test for categorical variables. Region-specific analyses were conducted using Rutherford MRI scores dichotomized as 0 (normal) or 1 (any injury).

To evaluate the discriminatory performance of the individual HRV metrics, we calculated the area under the receiver operating characteristic curve (AUC-ROC) at three time points. Optimal cut-offs were determined using the Youden Index.

Separate binary logistic regression models were constructed for each brain region to assess independent associations between the selected HRV metrics and regional injury. HRV variables were selected on the basis of (1) biological plausibility, (2) univariate *p* < 0.25, and (3) AUC-ROC performance. All models were adjusted for potential confounders, such as asphyxia severity, Sarnat stage, and presence of electroclinical seizures. Model selection was guided by Akaike information criterion (AIC), Bayesian information criterion (BIC), log-likelihood, and McFadden’s pseudo-R^2^.

Post hoc power analysis. To contextualize the precision of the region-specific results, we conducted Monte Carlo simulations (5,000 iterations; α = 0.05) for each topographic MRI outcome (BG/T, SB, cortex, and PLIC). We estimated the power to detect discriminatory effects corresponding to the prespecified AUC values (0.75 and 0.65) using the Wilcoxon rank-sum test. Logistic regression power was also simulated for an odds ratio of 2.0 per 1-SD predictor, with the model intercept calibrated to the observed prevalence of abnormal findings in each region. The detailed results are presented in Supplementary Table S15.

Statistical analyses were conducted using the R software version 4.4 (R Foundation for Statistical Computing, Vienna, Austria), and statistical significance was set at two-tailed *p* < 0.05.

## 3. Results

**Figure 1:**
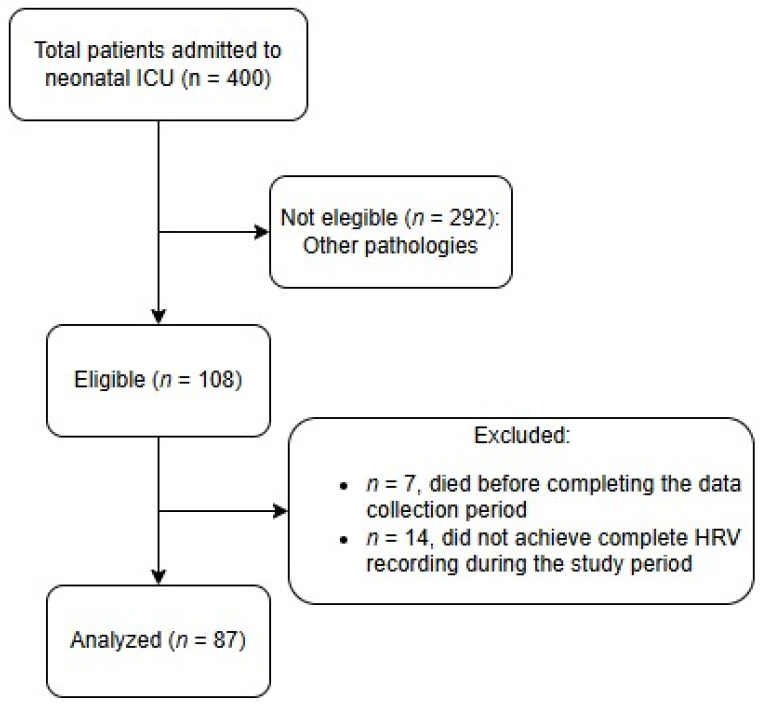
Study flow diagram.

Eighty-seven neonates were included in the study (Figure 1), of which 58 (66.7%) showed brain lesions on MRI. Seizures during TH were significantly more frequent in patients with cerebral injury than in those without cerebral injury (78.6% vs. 21.4%, *p* = 0.002; Table 1).

The distribution of injuries by region (Rutherford scoring system) is presented in Supplementary Table S1. BG/T and PLIC had the highest frequencies of moderate/severe lesions (BG/T, 16.1%); PLIC (12.6%), with additional mild injuries in both regions.

### 3.1. Predictive Capacity

As shown in Table 2, the LF and HF powers demonstrated significant discriminatory capacity. During the first 24 h, LF had an AUC of 0.79 (95% CI: 0.69–0.88), HF an AUC of 0.80 (95% CI: 0.72–0.89). Performance improved during rewarming: LF AUC = 0.90 (95% CI: 0.84–0.97), HF AUC = 0.82 (95% CI: 0.74–0.91). The LF cutoff of 67.8 ms^2^ (Youden Index) yielded 79.3% sensitivity and 96.6% specificity. The cut-offs for all HRV metrics are listed in Supplementary Table S2. In contrast, time-domain metrics such as SDNN and rMSSD showed no meaningful discrimination, with AUC values consistently around 0.50 across phases. Similarly, the LF/HF ratio demonstrated only modest predictive ability (AUC = 0.60). 

In region-specific analyses (Supp. Tables S3–S6), LF during rewarming predicted injury in BG/T (AUC = 0.87), white matter (0.85), cortex (0.78), and PLIC (0.84). Other metrics, such as rMSSD, pNN50, and LF/HF, showed acceptable predictive values (AUC ≥ 0.70) in selected regions and time points.

### 3.2. Association Between HRV and Cerebral MRI Abnormalities

LF and HF powers were significantly lower in neonates with MRI abnormalities during the first 24 h and the rewarming phases (*p* < 0.001). Conversely, the LF/HF ratio was significantly higher in neonates with normal MRI findings during the postrewarming period (*p* = 0.009; Table 3).

In the region-specific analysis based on the Rutherford scoring system, the frequency-domain parameters showed the most consistent associations. In neonates with basal ganglia and/or thalamic (BG/T) injury, both LF and HF power were significantly reduced during the first 24 h and rewarming phases (Supplementary Table S7). For white matter injury, LF power was lower during the first 24 h, and both LF and HF powers were reduced during rewarming (Supplementary Table S8). Cortical injury was associated with reduced LF power during the first 24 h of rewarming, and decreased HF power during rewarming (Supplementary Table S9). In neonates with PLIC injury, both LF and HF power significantly decreased across all three phases: the first 24 h, rewarming, and post-rewarming (Supplementary Table S10).

### 3.3. Multivariate Analysis

In the multivariate analysis (Table 4), higher HF power during the first 24 h was associated with lower odds of brain injury on MRI (aOR = 0.91 per unit increase, 95% CI: 0.84–0.99, *p* = 0.05), while lower LF power during rewarming was independently associated with reduced odds of injury (aOR = 0.93 per unit increase, 95% CI: 0.89–0.98, *p* = 0.01). Conversely, the presence of seizures was strongly associated with MRI-detected injuries (aOR = 8.71, 95% CI: 1.21–62.52, *p* = 0.03).

In region-specific models (Table 5), LF during rewarming was significantly associated with white matter injury (aOR = 0.96, 95% CI: 0.93–0.98, *p* = 0.04) and showed borderline associations with cortical and BG/T injuries. Severe asphyxia and seizures were independently associated with PLIC injury (Supplementary Tables S11–S14).

Post hoc simulations (Table S15) confirmed that regional subgroup analyses were adequately powered to detect large effects (AUC ≥ 0.75 or OR ≈ 2.0) but underpowered for modest associations (AUC = 0.65), explaining some of the non-significant findings observed for time-domain HRV metrics and LF/HF ratios.

## 4. Discussion

This single-center study evaluated heart rate variability (HRV) in neonates with moderate-to-severe perinatal asphyxia treated with therapeutic hypothermia (TH) at a high-complexity referral hospital in a low- and middle-income country (LMIC). Initially, HRV metrics were analyzed in relation to global MRI outcomes (normal vs. abnormal). Both low-frequency (LF) power during the rewarming phase and high-frequency (HF) power within the first 24 h showed strong predictive performance for brain injury. Building on these findings, we conducted a more detailed region-specific analysis using the Rutherford MRI scoring system, which confirmed the discriminative capacity of LF and HF powers across multiple brain regions. Moreover, these frequency-domain metrics were independently associated with localized brain injury in multivariate models, reinforcing their potential as early physiological biomarkers of neurological injury in neonates with perinatal asphyxia.

In contrast, our findings indicate that the predictive capacity of HRV metrics, particularly LF and HF powers, substantially diminishes beyond the rewarming phase, likely reflecting physiological stabilization of the autonomic nervous system (ANS) as the acute phase of HIE and TH resolves, which reduces between-group differences and drives a convergence of HRV values [21,24]. While HRV remains a promising tool for early prognostication [22], its utility is limited by physiological normalization and heterogeneity in the literature [21,25]. Notably, lower LF power still predicted more severe MRI brain injury post-rewarming, suggesting that some signals persist, albeit with a reduced effect size [12]. Taken together, these observations underscore the value of HRV as a real-time biomarker, particularly during the early cooling/rewarming window [24], and we acknowledge that the smaller sample sizes in this later phase may have further limited the detection of modest associations.

Perinatal asphyxia initiates a cascade of neurological and systemic alterations that disrupt autonomic nervous system (ANS) function. Brain injury, particularly in subcortical structures and the brainstem, along with myocardial dysfunction, hemodynamic instability, systemic inflammation, and seizures, have been shown to significantly alter HRV patterns [21,26]. In the early stages of asphyxia, a surge in sympathetic activity is typically observed, as reflected by an increase in the LF power. However, in more severe cases, progressive neuronal damage results in autonomic dysregulation, characterized by a reduction in both the LF and HF components, indicating global suppression of ANS activity [27,28]. The severity, duration, and distribution of hypoxic–ischemic injury largely determines the extent of ANS dysfunction. Prolonged hypoxia tends to affect the cerebral cortex and sympathetic pathways, causing damage to the gray matter, brainstem nuclei, and cortical regions, ultimately culminating in autonomic failure [21,29,30,31]. These pathophysiological mechanisms support the use of HRV as a dynamic marker of neurocardiac regulation and as a noninvasive tool for assessing the severity and spatial distribution of hypoxic–ischemic brain injury.

Recently, Chen et al. demonstrated that reduced LF and HF power during TH was significantly associated with moderate-to-severe brain injury, as defined by the National Institute of Child Health and Human Development (NICHD) MRI scoring system in neonates with hypoxic–ischemic encephalopathy, with LF power persisting as an independent predictor [32]. While their analysis was limited to global classifications of injury severity, our study extends these findings by incorporating region-specific assessment using the Rutherford scoring system, which enables anatomical localization of HRV associations with distinct brain structures.

Several prior studies have consistently linked HRV suppression to adverse neurological outcomes in neonates with HIE undergoing TH. Decreased LF and HF powers have been associated with more severe injuries and subcortical involvement [18,21,22]. These findings are consistent with our results, which demonstrate region-specific associations between reduced LF power and injury to the basal ganglia/thalamus and the posterior limb of the internal capsule. Unlike studies that have applied global classifications of injury [21,32], our approach adds granularity through region-specific analyses based on the Rutherford scoring system. This supports the notion that HRV disturbances are not only markers of global injury but also reflect subcortical vulnerability.

Recently, Goswami et al. demonstrated that decreased HRV, particularly lower DFA α1 values, were significantly associated with basal ganglia-predominant and mixed patterns of brain injury in neonates with HIE undergoing TH [24]. While their analysis focused on nonlinear HRV metrics and categorized injuries based on predominant topographic patterns, our study expanded on this by using the Rutherford scoring system to perform a region-specific analysis. We observed similar reductions in frequency-domain HRV metrics, particularly LF power, associated with injury to the basal ganglia/thalamus and the posterior limb of the internal capsule, reinforcing the sensitivity of HRV to subcortical brain injury and its potential as a physiological biomarker in this population. 

In addition to spectral indices, several studies have evaluated nonlinear HRV metrics, such as detrended fluctuation analysis (DFA α1), entropy-based measures, and Poincaré-derived indices, which capture signal complexity and long-range correlations and may provide complementary prognostic information [24]. Nevertheless, nonlinear metrics typically demand longer artifact-free recordings, stricter preprocessing, and parameter tuning, and remain less standardized across bedside platforms, which can limit reproducibility and clinical uptake. In contrast, frequency-domain measures (LF, HF, LF/HF) are widely supported by existing monitors, can be computed from shorter windows with straightforward preprocessing, and are easier to standardize and interpret. These practical advantages make spectral HRV particularly feasible for implementation in low- and middle-income settings, where resource constraints and workflow considerations favor robust, interpretable, and readily deployable methods. Moreover, recent global surveys indicate that clinicians in LMICs are highly engaged in applying therapeutic hypothermia protocols and frequently adopt adjunctive neuromonitoring tools such as aEEG and MRI, even more often than in some high-income settings, reflecting a readiness to incorporate accessible physiological biomarkers such as HRV [33].

Global findings align with previous evidence suggesting that HRV metrics, particularly LF during the rewarming phase and HF in the first 24 h, are associated with brain lesions on MRI in neonates with perinatal asphyxia. This may reflect a critical period of autonomic recovery and reorganization, during which physiological signals, such as HRV, more accurately reflect the underlying brain injury. However, unlike studies that reported predictive values across multiple time points (24 h to 7 days), we found no significant associations beyond the first 24 h and the rewarming phase. While prior research has identified SDNN, RMSSD, and LF/HF ratio as relevant indicators, our analysis did not confirm their predictive capacity. Some studies reported that RMSSD exhibited strong predictive power for brain injury severity [32], while others found that reduced LF power at 24 and 80 h was linked to adverse outcomes [34]. Discrepancies in the LF/HF ratio associations have also been reported, with some linking higher values to improved outcomes and others suggesting the opposite [21,22]. These inconsistencies may reflect differences in population characteristics, asphyxia severity, or methodological variations in the HRV acquisition and analysis.

Given the potential of HRV as a real-time, accessible biomarker, its implementation in LMICs could be particularly valuable as advanced neuromonitoring is often unavailable. Notably, none of the studies included in recent systematic reviews have assessed HRV in neonates with HIE from LMICs, underscoring a critical gap in the literature [22,23]. Tools such as HRV monitoring may support timely clinical interventions and tailored neuroprotective strategies, ultimately enhancing clinical decision making and long-term outcomes. Campbell et al. reinforced the concept of HRV not only as a marker of injury severity but also as a dynamic tool for tracking disease progression and guiding real-time management [35]. This aligns with our study’s broader goal of exploring feasible and innovative strategies for strengthening neuroprotective care in resource-limited settings.

This study has several strengths, including its prospective design, rigorous HRV signal-processing methodology, and the use of brain MRI as an objective outcome measure of injury. Moreover, it offers novel insights into the predictive capacity of HRV in neonates undergoing therapeutic hypothermia in low- and middle-income countries, thereby addressing a critical gap in the existing literature.

Despite these strengths, this study has several limitations that must be acknowledged. The sample size was relatively small, which may limit the generalizability of the findings. Subgroup analyses by brain region were limited by the small sample sizes (10–23 abnormal cases per region). Post hoc simulations indicated adequate power to detect large discriminatory effects (AUC ≥ 0.75), but limited power (<40–60%) for modest effects (AUC = 0.65). Accordingly, non-significant findings for certain HRV metrics in regional models should be interpreted as inconclusive rather than as a definitive absence of association. Although HRV provides valuable information on autonomic function, it remains an indirect biomarker of neurological injury and its interpretation can be influenced by multiple physiological and technical factors. Future research should include larger cohorts and adopt standardized HRV measurement and analysis protocols to validate its clinical utility. In LMICs, further studies should focus on long-term outcomes, including mortality and neurodevelopmental trajectories, to comprehensively assess the efficacy of therapeutic hypothermia and role of HRV-based neuromonitoring in improving neonatal care.

## 5. Conclusions

In conclusion, this exploratory study provides evidence that HRV metrics, particularly low-frequency (LF) power during rewarming and high-frequency (HF) power within the first 24 h, are associated with region-specific brain injury in neonates with moderate-to-severe perinatal asphyxia treated with therapeutic hypothermia. Using the Rutherford scoring system, we anatomically localized these associations, with the strongest predictive value observed for injuries involving the basal ganglia/thalamus and posterior limb of the internal capsule. These findings support the potential role of HRV as a physiological biomarker of subcortical brain injury, contributing to early risk stratification and informed clinical decision making. This study also highlights the need to develop and validate HRV-based neuromonitoring strategies in low- and middle-income countries, where access to advanced neuroimaging and intensive care is limited. Future research should confirm these associations in larger multicenter cohorts, explore the integration of HRV into scalable neuroprotective strategies, and incorporate long-term neurodevelopmental follow-ups to validate the prognostic utility of HRV beyond the neonatal period.

## Figures and Tables

**Table 1:** Clinical characteristics of neonates with normal versus abnormal brain MRI findings.

	Total Cohort		Normal MRI		Abnormal MRI		*p*-Value
Sex, *n* (%)							
Female	36	(41.40)	26	(72.20)	10	(27.80)	0.35
Male	51	(58.60)	32	(62.70)	19	(37.30)	
Gestational age (Ballard), *n* (%)							
≥37	77	(88.50)	53	(68.80)	24	(31.20)	0.29 ^1^
<37	10	(11.50)	5	(50.00)	5	(50.00)	
Birth weight, g, *n* (%)							
≥2.500	74	(85.10)	50	(67.60)	24	(32.40)	0.75 ^1^
<2.500	13	(14.90)	8	(61.50)	5	(38.50)	
Mode of delivery, *n* (%)							
Vaginal	45	(51.70)	29	(64.40)	16	(35.60)	0.64
Cesarean	42	(48.30)	29	(69.00)	13	(31.00)	
Admission temperature, *n* (%)							
33–35 °C	40	(50.60)	30	(68.20)	14	(31.80)	0.92
>35 °C	27	(31.00)	18	(66.70)	9	(33.30)	
<33 °C	16	(18.40)	10	(62.50)	6	(37.50)	
Apgar score at 1 min, *n* (%)							
≥5	54	(62)	36	(66.70)	18	(33.30)	1.00
<5	33	(38)	22	(66.70)	11	(33.30)	
Apgar Score at 5 min, *n* (%)							
≥5	87	(100)	58	(67)	29	(33.30)	
<5	-		-		-		
Severity of asphyxia, *n* (%)							
Moderate	37	(42.50)	26	(70.30)	11	(29.70)	0.54
Severe	50	(57.40)	32	(64.00)	18	(36.00)	
Sarnat classifications, *n* (%)							
Moderate	75	(86.20)	52	(69.30)	23	(30.70)	0.20 ^1^
Severe	12	(13.80)	6	(50.00)	6	(50.00)	
Umbilical Cord Blood Gases							
pH, median (IQR)	6.94	(0.10)	6.96	(0.10)	6.91	(0.17)	0.24 ^2^
HCO3, mean, (SD)	11.70	(5.10)	11.50	(5.03)	12.00	(5.05)	0.68
Base excess, median (IQR)	−18.00	(6.40)	−16.90	(6.70)	−18.00	(7.80)	0.30 ^2^
Lactate, mean (SD)	11.40	(4.30)	11.20	(4.50)	11.80	(3.90)	0.54
Clinical and/or Electrical Seizures, *n* (%)							
No	56	(64.40)	44	(78.60)	12	(21.4)	0.02 ^3^
Yes	31	(35.60)	14	(45.20)	17	(54.8)	
Inotropic support, *n* (%)							
No	36	(41.40)	23	(63.90)	13	(36.10)	0.64
Yes	51	(58.60)	35	(68.60)	16	(31.40)	

Abbreviations: MRI—magnetic resonance imaging; IQR—interquartile range; SD—standard deviation. ^1^ *p*-values were calculated using Fisher’s exact test for categorical variables.^2^ Mann–Whitney U test for continuous variables. ^3^ *p* < 0.05.

**Table 2:** Area Under the Curve (AUC) of Heart Rate Variability Metrics.

Time Point	AUC	95% CI		*p*-Value
First 24 h				
SDNN	0.53	0.41	0.66	0.62
rMSSD	0.55	0.42	0.67	0.47
pNN50	0.54	0.41	0.66	0.58
VLF power	0.51	0.38	0.64	0.93
LF power	0.79	0.69	0.88	<0.001 ^1^
HF power	0.80	0.72	0.89	<0.001 ^1^
LF/HF ratio	0.40	0.28	0.52	0.10
Rewarming phase				
SDNN	0.51	0.37	0.64	0.90
rMSSD	0.50	0.37	0.63	0.95
pNN50	0.50	0.37	0.63	0.99
VLF power	0.52	0.38	0.66	0.76
LF power	0.90	0.84	0.97	<0.001 ^1^
HF power	0.82	0.74	0.91	<0.001 ^1^
LF/HF ratio	0.59	0.47	0.72	0.14
24 h Post-Rewarming				
SDNN	0.46	0.33	0.59	0.54
rMSSD	0.41	0.28	0.55	0.21
pNN50	0.38	0.26	0.51	0.06
VLF power	0.51	0.38	0.63	0.93
LF power	0.55	0.43	0.67	0.43
HF power	0.45	0.33	0.58	0.48
LF/HF ratio	0.67	0.56	0.79	<0.001 ^1^

SDNN: Standard deviation of all NN intervals; rMSSD: Root mean square of successive differences between consecutive NN intervals; pNN50: Percentage of NN intervals differing by more than 50 ms; VLF Power: Very-low-frequency power (<0.04 Hz); LF Power: Low-frequency power (0.04–0.15 Hz); HF Power: High-frequency power (0.15–0.4 Hz); LF/HF Ratio: Ratio between low-frequency and high-frequency power. ^1^ *p*-value < 0.05.

**Table 3:** Association Between Heart Rate Variability Metrics and Brain MRI Abnormalities in the First Week of Life.

	Brain MRI				
	Normal		Abnormal		
	Median	IQR	Median	IQR	*p*-Value ^1^
First 24 h					
SDNN					0.63
rMSSD	26	25	23	23	0.48
pNN50	5	20	4	14	0.59
VLF power	515.90	596.30	584.40	532.30	0.92
LF power	168.90	280.30	54.80	27.80	<0.001 ^2^
HF power	53.10	105.40	11.70	9	<0.001 ^2^
LF/HF ratio	3.20	4.1	4.1	3.7	0.10
Rewarming phase					
SDNN	30	20.50	29	29	0.90
rMSSD	22	18	20.50	19	0.95
pNN50	3.50	9	2	11	0.98
VLF power	429.90	476.30	411.70	879.90	0.74
LF power	237.10	314.40	40.50	28.40	<0.001 ^2^
HF power	78.60	105.40	10.90	18.90	<0.001 ^2^
LF/HF ratio	3.70	3.20	2.90	2.50	0.15
24 h Post-Rewarming					
SDNN	24	8	26	10	0.53
rMSSD	17.80	12	24.50	15	0.19
pNN50	1.80	7	5	8	0.06
VLF power	290.90	283.40	288.40	211	0.93
LF power	134.40	138.70	130.60	113.70	0.44
HF power	36.20	41.60	47.40	40.10	0.48
LF/HF ratio	3.85	2.79	2.90	1.45	0.009 ^2^

^1^ Mann–Whitney U test. SDNN: Standard deviation of all NN intervals; rMSSD: Root mean square of successive differences between consecutive NN intervals; pNN50: Percentage of NN intervals differing by more than 50 ms; VLF Power: Very-low-frequency power (<0.04 Hz); LF Power: Low-frequency power (0.04–0.15 Hz); HF Power: High-frequency power (0.15–0.4 Hz); LF/HF Ratio: Ratio between low-frequency and high-frequency power. ^2^ *p* < 0.05.

**Table 4:** Association Between HRV Metrics and brain MRI Abnormalities: Unadjusted and Adjusted Logistic Regression Models.

Variable	OR	95% CI		Valor p	aOR	95% CI		*p*-Value
High-Frequency Power (HF) in the First 24 h	0.93	0.90	0.97	0.002	0.91	0.84	0.99	0.05 ^1^
Low-Frequency Power (LF) During Rewarming	0.96	0.94	0.98	0.002	0.93	0.89	0.98	0.01 ^1^
High-Frequency Power (HF) During Rewarming	0.96	0.94	0.98	<0.001	1.04	0.98	1.10	0.14
Clinical/Electrical Seizures								
No (reference)								
Yes	6.05	2.25	16.26	<0.001	8.71	1.21	62.52	0.03 ^1^
Severity of Asphyxia								
Moderate (reference)								
Severe	1.32	0.53	3.30	0.64	1.18	0.20	6.75	0.84
Severity of Encephalopathy (Sarnat Classification)								
Moderate (reference)								
Severe	2.26	0.65	7.76	0.19	1.70	0.14	20.21	0.67

HRV: Heart rate variability; MRI: Magnetic resonance imaging; OR: Odds ratio; aOR: Adjusted odds ratio; CI: Confidence interval; LF power: Power in the low-frequency band (0.04–0.15 Hz); HF power: Power in the high-frequency band (0.15–0.4 Hz). Reference categories: Clinical/electrical seizures (No), Severity of asphyxia (Moderate), and Hypoxic–ischemic encephalopathy (HIE) severity according to Sarnat classification (Moderate). Adjusted models control perinatal asphyxia, HIE severity, and the presence of seizures. ^1^ A *p*-value < 0.05 was considered statistically significant.

**Table 5:** Adjusted Logistic Regression Models Evaluating the Association Between Heart Rate Variability Metrics and Region-Specific Brain Injury Based on Rutherford scoring system on Neonatal MRI During the First Week of Life.

Region	HRV Metric	Adjusted OR	95% CI	*p*-Value
Basal Ganglia/Thalamus (BG/T)	HF Power (First 24 h)	0.94	0.87–0.99	0.10
	LF Power (Rewarming)	0.97	0.94–0.99	0.08
	LF/HF Ratio (Post-Rewarming)	0.64	0.34–1.09	0.12
White Matter	LF Power (Rewarming)	0.96	0.93–0.98	0.04 ^1^
Cortex	LF Power (Rewarming)	0.97	0.94–0.99	0.08
Cortex	HF Power (Rewarming)	1.02	0.97–1.02	0.41
Posterior Limb of Internal Capsule (PLIC)	HF Power (post-rewarming)	1.00	0.98–1.02	0.83
	LF/HF Ratio (Post-Rewarming)	0.67	0.41–1.04	0.09

Abbreviations: HF = high-frequency power (0.15–0.4 Hz); LF = low-frequency power (0.04–0.15 Hz); LF/HF = ratio of LF to HF power. Adjusted odds ratios (OR), 95% confidence intervals (CI), and *p*-values were obtained from binary logistic regression models, each adjusted for perinatal asphyxia severity (moderate/severe), hypoxic–ischemic encephalopathy severity (Sarnat II/III), and presence of electroclinical seizures. One model was estimated for each brain region using the Rutherford scoring system. Complete unadjusted models and covariate-specific effects are reported in Supplementary Tables S9–S12. ^1^ A *p*-value < 0.05 was considered statistically significant.

## Data Availability

The data presented in this study are available on request from the corresponding author due to reasonable request.

## Supplementary Materials

The following supporting information can be downloaded from https://www.mdpi.com/article/10.3390/medicina61091631/s1, Table S1: Distribution of Brain Injury Severity by Anatomical Region According to the Rutherford scoring system; Table S2: HRV Metrics with Predictive Capacity (AUC) and Optimal Sensitivity/Specificity Values; Table S3: Discriminative Performance of Heart Rate Variability Metrics for Predicting Basal Ganglia and/or Thalamic Injury According to Rutherford Classification; Table S4: Discriminative Performance of Heart Rate Variability Metrics for Predicting White Matter Injury According to Rutherford Classification; Table S5: Discriminative Performance of Heart Rate Variability Metrics for Predicting Cortical Injury According to Rutherford Classification; Table S6: Discriminative Performance of Heart Rate Variability Metrics for Predicting Posterior Limb of the Internal Capsule Injury According to Rutherford Classification; Table S7: Association Between Heart Rate Variability Metrics and Basal Ganglia and/or Thalamic Injury (BG/T) according to Rutherford Classification During the First Week of Life; Table S8: Association Between Heart Rate Variability Metrics and White Matter Injury according to Rutherford Classification During the First Week of Life; Table S9: Association Between Heart Rate Variability Metrics and Cortical Injury according to Rutherford Classification During the First Week of Life; Table S10: Association Between Heart Rate Variability Metrics and Posterior Limb of the Internal Capsule (PLIC) according to Rutherford Classification During the First Week of Life; Table S11: Association Between Heart Rate Variability Metrics and Basal Ganglia and/or Thalamic Injury (BG/T) according to Rutherford Classification During the First Week of Life: Unadjusted and Adjusted Logistic Regression Models; Table S12: Association Between Heart Rate Variability Metrics and White Matter Injury on according to Rutherford Classification During the First Week of Life: Unadjusted and Adjusted Logistic Regression Models; Table S13: Association Between Heart Rate Variability Metrics and Cortical Injury according to Rutherford Classification During the First Week of Life: Unadjusted and Adjusted Logistic Regression Models; Table S14: Association Between Heart Rate Variability Metrics and Posterior Limb of the Internal Capsule (PLIC) Injury according to Rutherford Classification During the First Week of Life: Unadjusted and Adjusted Logistic Regression Models; Table S15: Post-hoc power for region-specific analyses.

## Abbreviations

The following abbreviations are used in this manuscript:

HRV	Heart Rate Variability
AUC	Area Under the Curve
CI	Confidence Interval
LF	Low-Frequency Power
HF	High-Frequency Power
SDNN	Standard deviation of all NN intervals
rMSSD	Root mean square of successive differences between consecutive NN intervals
pNN50	Percentage of NN intervals differing by more than 50 ms
VLF	Very-Low-Frequency Power
LF/HF Ratio	Ratio between low-frequency and high-frequency power
OR	Odds Ratios
BG/T	Basal Ganglia/Thalamus
PLIC	Posterior Limb of Internal Capsule
IQR	Interquartile Range
EHI	Hypoxic–Ischemic Encephalopathy
TH	Therapeutic Hypothermia
LMIC	Low- and middle-income country
