# Supplementary material for: Heart Rate Variability as a Predictor of Region-Specific Brain Injury in Neonates with Perinatal Asphyxia: A Prospective Study in a Middle-Income Country
Source: Medicina (Kaunas). 2025 Sep 9;61(9):1631. doi: 10.3390/medicina61091631 (PMC12471330; doi:10.3390/medicina61091631)
Supplement: Supplementary file 1 [file medicina-61-01631-s001.zip › medicina-3848201-supplementary.pdf]

Supplementary materials

Heart Rate Variability Predicts Region-Specific Brain Injury in Neonates with Perinatal Asphyxia: A Prospective Study in a Middle-Income Country

Sergio Agudelo-Pérez[1*;] Gloria Troncoso[2], Alvaro Arenas Aulí[3], Camila Ayala[4]

[1] PhD. Health of Sciences; Pediatric Neonatologist; Associate professor, Department of Pediatrics, School of Medicine, Universidad de La Sabana. Chía, Colombia. ORCID: https://orcid.org/0000-0001-9154-4529. Email: sergio.agudelo1@unisabana.edu.co

[2] Pediatric Neonatologist; Head Neonatal Unit, Fundación Cardioinfantil – Instituto de Cardiología, Bogotá, Colombia. ORCID: https://orcid.org/0000-0002-1980-5656. Email: gtroncoso@lacardio.org

[3] Pediatric Cardiologist and Electrophysiologist; Coordinator, Pediatric Electrophysiology, Fundación Cardioinfantil - Instituto de Cardiología, Bogotá, Colombia. ORCID: https://orcid.org/0000-0002-2208-1767. Email: aarenas@lacardio.org

[4] School of Medicine, Universidad de La Sabana, Chía, Colombia. ORCID: https://orcid.org/0009-0005-3890-2450. Email: camilaayago@unisabana.edu.co

* Corresponding author: Sergio Agudelo-Pérez. Universidad de La Sabana, Campus Puente del Común, Km. 7, Autopista Norte de Bogotá, Chía, Cundinamarca, Colombia. P.O. Box 53753, Bogotá. Email: sergio.agudelo1@unisabana.edu.co

Supplementary Table S1. Distribution of brain injury severity by anatomical region according to the Rutherford classification (n = 87)

Anatomical Region	Normal n (%)	Mild n (%)	Moderate/Severe n (%)	Total n (%)
Basal Ganglia / Thalamus (GB/T)	64 (73.60)	9 (10.30)	14 (16.10)	87 (100)
White Matter	77 (88.50)	8 (9.20)	2 (2.30)	87 (100)
Cortex	75 (86.20)	7 (8.00)	5 (5.70)	87 (100)
Posterior Limb of Internal Capsule (PLIC)	70 (80.50)	6 (6.90)	11 (12.60)	87 (100)

Brain injury severity was classified based on the original Rutherford scale: 0 = normal; 1 = mild injury; 2–3 = moderate to severe injury. Each region was evaluated independently based on standard neonatal brain MRI sequences

Supplementary Table S2. HRV Metrics with Predictive Capacity (AUC) and Optimal Sensitivity/Specificity Values

HRV Metric	Optimal Cutoff Value	AUC (95% CI)	Sensitivity	Specificity
Low-Frequency Power (First 24h)	76.30	0.79 (0.69–0.88)	74.10%	89.00%
High-Frequency Power (First 24h)	19.70	0.80 (0.72–0.89)	74.10%	86.20%
Low-Frequency Power (Rewarming)	67.80	0.90 (0.84–0.97)	79.30%	96.60%
High-Frequency Power (Rewarming)	40.40	0.82 (0.74–0.91)	70.70%	93.10%

HRV: Heart rate variability; AUC: Area under the curve; CI: Confidence interval; LF power: Low-frequency power (0.04–0.15 Hz); HF: High-frequency power (0.15–0.4 Hz). Sensitivity and specificity values correspond to the optimal cutoff determined by maximizing the Youden Index.

Supplementary Table S3. Discriminative Performance of Heart Rate Variability Metrics for Predicting Basal Ganglia and/or Thalamic Injury According to the Rutherford Classification

Time point	AUC	95% CI	
First 24 hours			
SDNN	0.54	0.40	0.67
rMSSD	0.50	0.37	0.67
pNN50	0.48	0.36	0.61
VLF power	0.46	0.32	0.61
LF power	**0.78**	**0.69**	**0.88**
HF power	**0.78**	**0.68**	**0.87**
LF/HF ratio	0.45	0.31	0.58
Rewarming phase			
SDNN	0.52	0.37	0.67
rMSSD	0.50	0.36	0.65
pNN50	0.50	0.36	0.64
VLF power	0.54	0.38	0.70
LF power	**0.87**	**0.79**	**0.94**
HF power	**0.81**	**0.73**	**0.90**
LF/HF ratio	0.57	0.43	0.70
24 Hours Post-Rewarming			
SDNN	0.58	0.44	0.73
rMSSD	0.61	0.47	0.76
pNN50	0.62	0.48	0.75
VLF power	0.52	0.38	0.67
LF power	0.47	0.33	0.61
HF power	0.58	0.44	0.71
LF/HF ratio	**0.71**	**0.60**	**0.82**

SDNN: Standard deviation of all NN intervals; rMSSD: Root mean square of successive differences between consecutive NN intervals; pNN50: Percentage of NN intervals differing by more than 50 ms; VLF Power: Very-low-frequency power (<0.04 Hz); LF Power: Low-frequency power (0.04–0.15 Hz); HF Power: High-frequency power (0.15–0.4 Hz); LF/HF Ratio: Ratio between low-frequency and high-frequency power.

Supplementary Table S4. Discriminative Performance of Heart Rate Variability Metrics for Predicting White Matter Injury According to the Rutherford Classification

Time point	AUC	95% CI	
First 24 hours			
SDNN	0.55	0.37	0.74
rMSSD	0.60	0.40	0.81
pNN50	0.59	0.39	0.79
VLF power	0.51	0.30	0.71
LF power	0.68	0.55	0.80
HF power	**0.72**	**0.60**	**0.84**
LF/HF ratio	0.62	0.50	0.75
Rewarming phase			
SDNN	0.60	0.38	0.82
rMSSD	0.59	0.38	0.80
pNN50	0.54	0.33	0.75
VLF power	0.56	0.34	0.78
LF power	**0.85**	**0.76**	**0.93**
HF power	**0.74**	**0.62**	**0.86**
LF/HF ratio	0.60	0.39	0.81
24 Hours Post-Rewarming			
SDNN	0.65	0.48	0.83
rMSSD	0.57	0.37	0.76
pNN50	0.51	0.34	0.67
VLF power	0.63	0.47	0.78
LF power	0.64	0.46	0.81
HF power	0.63	0.45	0.81
LF/HF ratio	0.55	0.35	0.75

SDNN: Standard deviation of all NN intervals; rMSSD: Root mean square of successive differences between consecutive NN intervals; pNN50: Percentage of NN intervals differing by more than 50 ms; VLF Power: Very-low-frequency power (<0.04 Hz); LF Power: Low-frequency power (0.04–0.15 Hz); HF Power: High-frequency power (0.15–0.4 Hz); LF/HF Ratio: Ratio between low-frequency and high-frequency power.

Supplementary Table S5. Discriminative Performance of Heart Rate Variability Metrics for Predicting Cortical Injury According to the Rutherford Classification

Time point	AUC	95% CI	
First 24 hours			
SDNN	0.54	0.33	0.74
rMSSD	0.58	0.38	0.77
pNN50	0.57	0.38	0.76
VLF power	0.47	0.29	0.66
LF power	**0.72**	**0.60**	**0.84**
HF power	**0.70**	**0.58**	**0.83**
LF/HF ratio	0.49	0.34	0.63
Rewarming phase			
SDNN	0.55	0.35	0.76
rMSSD	0.55	0.37	0.72
pNN50	0.58	0.44	0.73
VLF power	0.58	0.37	0.79
LF power	**0.78**	**0.68**	**0.88**
HF power	**0.73**	**0.61**	**0.85**
LF/HF ratio	0.51	0.35	0.68
24 Hours Post-Rewarming			
SDNN	0.63	0.46	0.81
rMSSD	0.65	0.47	0.82
pNN50	0.65	0.47	0.82
VLF power	0.58	0.38	0.77
LF power	0.48	0.31	0.64
HF power	0.62	0.46	0.77
LF/HF ratio	0.65	0.51	0.79

SDNN: Standard deviation of all NN intervals; rMSSD: Root mean square of successive differences between consecutive NN intervals; pNN50: Percentage of NN intervals differing by more than 50 ms; VLF Power: Very-low-frequency power (<0.04 Hz); LF Power: Low-frequency power (0.04–0.15 Hz); HF Power: High-frequency power (0.15–0.4 Hz); LF/HF Ratio: Ratio between low-frequency and high-frequency power.

Supplementary Table S6. Discriminative Performance of Heart Rate Variability Metrics for Predicting Posterior Limb of the Internal Capsule Injury According to the Rutherford Classification

Time point	AUC	95% CI	
First 24 hours			
SDNN	0.53	0.37	0.69
rMSSD	0.54	0.40	0.68
pNN50	0.56	0.43	0.70
VLF power	0.53	0.36	0.70
LF power	**0.79**	**0.69**	**0.89**
HF power	**0.78**	**0.68**	**0.88**
LF/HF ratio	0.47	0.31	0.63
Rewarming phase			
SDNN	0.50	0.33	0.68
rMSSD	0.47	0.31	0.62
pNN50	0.46	0.32	0.61
VLF power	0.49	0.31	0.68
LF power	**0.84**	**0.76**	**0.92**
HF power	**0.80**	**0.70**	**0.90**
LF/HF ratio	0.54	0.38	0.69
24 Hours Post-Rewarming			
SDNN	0.64	0.49	0.79
rMSSD	0.65	0.50	0.80
pNN50	0.64	0.49	0.79
VLF power	0.61	0.46	0.75
LF power	0.56	0.41	0.70
HF power	0.63	0.48	0.77
LF/HF ratio	0.67	0.55	0.79

SDNN: Standard deviation of all NN intervals; rMSSD: Root mean square of successive differences between consecutive NN intervals; pNN50: Percentage of NN intervals differing by more than 50 ms; VLF Power: Very-low-frequency power (<0.04 Hz); LF Power: Low-frequency power (0.04–0.15 Hz); HF Power: High-frequency power (0.15–0.4 Hz); LF/HF Ratio: Ratio between low-frequency and high-frequency power.

Supplementary Table S7. Association Between Heart Rate Variability Metrics and Basal Ganglia and/or Thalamic Injury according to the Rutherford Classification During the First Week of Life

Variable	Normal Median (IQR)	Abnormal Median (IQR)	p-value
First 24 hours			
SDNN	33.00 (19.00)	32.00 (12.00)	0.77
rMSSD	24.00 (26.00)	23.00 (24.00)	0.99
pNN50	4.50 (21.00)	4.0 (15.00)	0.85
VLF power	523.40 (566.50)	572.00 (468.30)	0.63
LF power	141.7 (265.70)	54.80 (30.10)	**<0.001**
HF power	49.80 (105.30)	11.70 (9.50)	**<0.001**
LF/HF ratio	3.80 (4.00)	3.60 (3.30)	0.47
Rewarming phase			
SDNN	30.00 (19.10)	29.00 (28.90)	0.75
rMSSD	22.000 (18.30)	20.00 (19.50)	0.96
pNN50	3.5 (9.00)	2.00 (10.00)	0.97
VLF power	429.90 (494.70)	337.20 (763.50)	0.57
LF power	217.00 (310.60)	41.00 (26.20)	**<0.001**
HF power	70.10 (104.00)	9.30 (21.50)	**<0.001**
LF/HF ratio	3.50 (2.90)	3.30 (2.50)	0.35
24 Hours Post-Rewarming			
SDNN	24.00 (8.00)	27.00 (9.60)	0.23
rMSSD	17.80 (12.60)	26.0 (14.50)	0.11
pNN50	2.00 (7.00)	5.0 (9.00)	0.09
VLF power	285.40 (264.80)	309.8 (342.70)	0.77
LF power	129.70 (126.70)	132.9 (134.90)	0.66
HF power	36.20 (39.70)	49.1 (33.30)	0.27
LF/HF ratio	3.80 (2.60)	2.7 (1.10)	**<0.001**

*Mann-Whitney U test.

SDNN: Standard deviation of all NN intervals; rMSSD: Root mean square of successive differences between consecutive NN intervals; pNN50: Percentage of NN intervals differing by more than 50 ms; VLF Power: Very-low-frequency power (<0.04 Hz); LF Power: Low-frequency power (0.04–0.15 Hz); HF Power: High-frequency power (0.15–0.4 Hz); LF/HF Ratio: Ratio between low-frequency and high-frequency power.

Supplementary Table S8. Association Between Heart Rate Variability Metrics and White Matter Injury according to the Rutherford Classification During the First Week of Life

Variable	Normal<br>Median (IQR)	Abnormal<br>Median (IQR)	p-value*
First 24 hours			
SDNN	33.00 (19.00)	32.50 (17.25)	0.58
rMSSD	25.00 (25.00)	18.50 (26.00)	0.29
pNN50	5.00 (20.00)	2.50 (16.50)	0.37
VLF power	526.70 (504.80)	494.25 (566.33)	0.95
LF power	114.00 (244.29)	58.05 (23.67)	0.07
HF power	27.50 (92.00)	13.90 (7.53)	**0.02**
LF/HF ratio	3.38 (4.06)	4.25 (1.83)	0.20
Rewarming phase			
SDNN	30.00 (19.00)	35.75 (22.00)	0.30
rMSSD	21.00 (16.00)	26.75 (24.50)	0.34
pNN50	3.00 (9.00)	4.00 (4.00)	0.68
VLF power	424.90 (518.80)	498.15 (752.13)	0.55
LF power	174.00 (289.65)	32.95 (28.93)	**<0.001**
HF power	58.90 (99.60)	14.20 (14.85)	**0.02**
LF/HF ratio	3.53 (2.91)	2.81 (2.25)	0.32
24 Hours Post-Rewarming			
SDNN	25.00 (8.50)	20.50 (6.88)	0.12
rMSSD	21.00 (14.00)	18.00 (12.50)	0.49
pNN50	2.00 (8.00)	3.00 (5.13)	0.96
VLF power	300.10 (291.65)	231.60 (66.72)	0.19
LF power	134.50 (127.00)	83.85 (83.85)	0.16
HF power	42.80 (40.50)	23.45 (32.33)	0.19
LF/HF ratio	3.50 (2.30)	2.93 (1.88)	0.63

*Mann-Whitney U test.

SDNN: Standard deviation of all NN intervals; rMSSD: Root mean square of successive differences between consecutive NN intervals; pNN50: Percentage of NN intervals differing by more than 50 ms; VLF Power: Very-low-frequency power (<0.04 Hz); LF Power: Low-frequency power (0.04–0.15 Hz); HF Power: High-frequency power (0.15–0.4 Hz); LF/HF Ratio: Ratio between low-frequency and high-frequency power.

Supplementary Table S9. Association Between Heart Rate Variability Metrics and Cortical Injury according to the Rutherford Classification During the First Week of Life

Variable	Normal<br>Median (IQR)	Abnormal<br>Median (IQR)	p-value*
First 24 hours			
SDNN	33.00 (18.00)	32.00 (22.25)	0.68
rMSSD	25.00 (24.50)	16.00 (25.00)	0.40
pNN50	5.00 (19.00)	1.50 (15.00)	0.45
VLF power	520.00 (552.65)	578.20 (412.40)	0.76
LF power	114.70 (247.45)	60.58 (33.73)	**0.02**
HF power	28.50 (96.60)	14.40 (10.03)	**0.03**
LF/HF ratio	3.96 (4.08)	3.26 (1.90)	0.88
Rewarming phase			
SDNN	30.00 (21.25)	31.25 (26.25)	0.57
rMSSD	22.00 (19.00)	20.00 (8.50)	0.61
pNN50	4.00 (10.50)	2.00 (4.50)	0.35
VLF power	411.70 (517.80)	574.55 (1064.08)	0.37
LF power	179.90 (291.93)	44.38 (25.19)	**<0.001**
HF power	58.90 (99.65)	12.75 (20.20)	**0.01**
LF/HF ratio	3.51 (2.92)	3.25 (2.25)	0.88
24 Hours Post-Rewarming			
SDNN	24.00 (8.00)	28.00 (7.00)	0.14
rMSSD	18.00 (13.25)	23.50 (15.50)	0.11
pNN50	2.00 (7.00)	5.50 (12.75)	0.10
VLF power	285.80 (269.30)	293.20 (424.63)	0.40
LF power	128.80 (134.10)	133.70 (114.84)	0.82
HF power	37.40 (39.53)	52.35 (21.59)	0.19
LF/HF ratio	3.60 (2.65)	2.93 (0.99)	0.10

*Mann-Whitney U test.

SDNN: Standard deviation of all NN intervals; rMSSD: Root mean square of successive differences between consecutive NN intervals; pNN50: Percentage of NN intervals differing by more than 50 ms; VLF Power: Very-low-frequency power (<0.04 Hz); LF Power: Low-frequency power (0.04–0.15 Hz); HF Power: High-frequency power (0.15–0.4 Hz); LF/HF Ratio: Ratio between low-frequency and high-frequency power.

Supplementary Table S10. Association Between Heart Rate Variability Metrics and Posterior Limb of the Internal Capsule (PLIC) according to the Rutherford Classification During the First Week of Life

Variable	Normal Median (IQR)	Abnormal Median (IQR)	p-value*
First 24 hours			
SDNN	32.00 (18.50)	34.00 (14.00)	0.69
rMSSD	23.00 (25.75)	29.00 (23.00)	0.59
pNN50	3.50 (20.50)	10.50 (13.00)	0.42
VLF power	515.95 (561.45)	614.90 (458.70)	0.71
LF power	122.65 (259.20)	47.41 (42.43)	**<0.001**
HF power	37.55 (101.28)	11.30 (8.40)	**<0.001**
LF/HF ratio	3.77 (3.98)	3.49 (4.19)	0.67
Rewarming phase			
SDNN	30.00 (20.63)	29.00 (30.00)	0.95
rMSSD	22.00 (18.25)	20.50 (17.00)	0.66
pNN50	3.00 (9.00)	2.00 (10.00)	0.64
VLF power	423.40 (475.83)	488.10 (920.80)	0.95
LF power	200.85 (292.08)	41.00 (29.65)	**<0.001**
HF power	65.05 (101.18)	8.20 (20.60)	**<0.001**
LF/HF ratio	3.52 (2.85)	3.32 (3.14)	0.65
24 Hours Post-Rewarming			
SDNN	24.00 (8.00)	28.00 (6.25)	0.07
rMSSD	17.75 (12.88)	27.00 (14.00)	**0.05**
pNN50	2.00 (7.00)	7.00 (10.00)	0.06
VLF power	281.30 (273.93)	360.40 (242.70)	0.18
LF power	127.25 (136.50)	144.90 (94.70)	0.47
HF power	36.20 (38.98)	49.10 (47.50)	0.11
LF/HF ratio	3.75 (2.50)	2.90 (0.90)	**0.03**

*Mann-Whitney U test.

SDNN: Standard deviation of all NN intervals; rMSSD: Root mean square of successive differences between consecutive NN intervals; pNN50: Percentage of NN intervals differing by more than 50 ms; VLF Power: Very-low-frequency power (<0.04 Hz); LF Power: Low-frequency power (0.04–0.15 Hz); HF Power: High-frequency power (0.15–0.4 Hz); LF/HF Ratio: Ratio between low-frequency and high-frequency power.

Supplementary Table S11. Association Between Heart Rate Variability Metrics and Basal Ganglia and/or Thalamic Injury (GB/T) according to the Rutherford Classification During the First Week of Life: Unadjusted and Adjusted Logistic Regression Models

Variable	Unadjusted OR	95% CI		p-value	Adjusted OR	95% CI		p-value
High-Frequency Power (First 24h)	0.94	0.89	0.97	**0.008**	0.94	0.87	0.99	0.10
Low-Frequency Power (Rewarming)	0.97	0.95	0.98	**0.004**	0.97	0.94	0.99	0.08
LF/HF ratio (post-rewarming)	0.59	0.39	0.82	**0.004**	0.64	0.34	1.09	0.12
Severity of asphyxia								
Moderate				Reference				
Severe	2.02	0.75	5.86	0.17	1.80	0.36	9.35	0.47
Encephalopathy hypoxic-Ischemic severity (SARNAT)								
Moderate (Sarnat II)				Reference				
Severe (Sarnat II)	1.47	0.36	5.26	0.56	0.93	0.09	9.95	0.95
Electroclinical Seizures								
No				Reference				
Yes	7.36	2.64	22.10	**<0.001**	7.06	1.57	40.93	**0.02**

HF Power: High-frequency power (0.15–0.4 Hz); LF Power: Low-frequency power (0.04–0.15 Hz); LF/HF Ratio: Ratio between low-frequency and high-frequency power.

Unadjusted and adjusted odds ratios (OR) with 95% confidence intervals (CI) and p-values were estimated using binary logistic regression models. Adjusted models include perinatal asphyxia severity (moderate/severe), hypoxic-ischemic encephalopathy severity (Sarnat II/III), and the presence of electroclinical seizures.

Supplementary Table S12. Association Between Heart Rate Variability Metrics and White Matter Injury according to the Rutherford Classification During the First Week of Life: Unadjusted and Adjusted Logistic Regression Models

Variable	Unadjusted OR	95% CI		p-value	Adjusted OR	95% CI		p-value
Low-Frequency Power (Rewarming)	0.97	0.93	0.99	0.04	0.96	0.93	0.98	0.04
Severity of asphyxia								
Moderate	Reference							
Severe	0.45	0.10	1.70	0.24	0.21	0.03	1.01	0.06

LF Power: Low-frequency power (0.04–0.15 Hz). Unadjusted and adjusted odds ratios (OR) with 95% confidence intervals (CI) and p-values were estimated using binary logistic regression models. Adjusted models include perinatal asphyxia severity (moderate/severe), hypoxic-ischemic encephalopathy severity (Sarnat II/III), and the presence of electroclinical seizures. The moderate category was used as reference.

Supplementary Table S13. Association Between Heart Rate Variability Metrics and Cortical Injury according to the Rutherford Classification During the First Week of Life: Unadjusted and Adjusted Logistic Regression Models

Variable	Unadjusted OR	95% CI		p-value	Adjusted OR	95% CI		p-value
Low-Frequency Power (Rewarming)	0.98	0.96	0.99	**0.03**	0.97	0.94	0.99	0.08
High-Frequency Power (Rewarming)	0.97	0.94	0.99	**0.03**	1.02	0.97	1.02	0.41
Severity of asphyxia								
Moderate				Reference				
Severe	1.04	0.30	3.80	0.94	0.69	0.16	2.99	0.61
Electroclinical Seizures								
No				Reference				
Yes	3.60	1.04	13.40	**0.04**	2.14	0.54	8.95	0.27

HF Power: High-frequency power (0.15–0.4 Hz); LF Power: Low-frequency power (0.04–0.15 Hz).
Unadjusted and adjusted odds ratios (OR) with 95% confidence intervals (CI) and p-values were estimated using binary logistic regression models.
Adjusted models include perinatal asphyxia severity (moderate vs. severe), hypoxic-ischemic encephalopathy severity (Sarnat II/III), and the presence of electroclinical seizures.

Supplementary Table S14. Association Between Heart Rate Variability Metrics and Posterior Limb of the Internal Capsule (PLIC) Injury according to the Rutherford Classification During the First Week of Life: Unadjusted and Adjusted Logistic Regression Models

Variable	Unadjusted OR	95% CI		p-value	Adjusted OR	95% CI		p-value
High-Frequency Power (post-rewarming)	1.01	0.99	1.02	0.29	1.00	0.98	1.02	0.83
LF/HF ratio (post-rewarming)	0.68	0.46	0.97	**0.04**	0.67	0.41	1.04	0.09
Severity of asphyxia								
Moderate					Reference			
Severe	16.90	3.19	314	**0.007**	20.60	3.27	418.00	**0.01**
Electroclinical Seizures								
No					Reference			
Yes	5.72	1.89	18.90	**0.002**	6.68	1.90	27.20	**0.004**

HF Power: High-frequency power (0.15–0.4 Hz); LF/HF Ratio: Ratio between low-frequency (0.04–0.15 Hz) and high-frequency power.

Unadjusted and adjusted odds ratios (OR) with 95% confidence intervals (CI) and p-values were estimated using binary logistic regression models.

Adjusted models include perinatal asphyxia severity (moderate/severe) and presence of electroclinical seizures.

Table S15. Post-hoc power for region-specific analyses

Region	n cases	n controls	Power (AUC=0.75)	Power (AUC=0.65)	Power (OR=2.0)
GB/T (basal ganglia/thalamus)	23	64	0.97	0.58	0.74
SB (white matter)	10	77	0.77	0.34	0.48
Cortex	12	75	0.83	0.39	0.53
PLIC (posterior limb of internal capsule)	17	70	0.92	0.49	0.66

Post-hoc power was estimated via Monte Carlo simulation (5,000 iterations; $\alpha=0.05$). For AUC, case/control scores were generated from normal distributions separated to match a prespecified “true” AUC (0.75 or 0.65), and separation was tested with the Wilcoxon rank-sum test. For logistic regression, a standard-normal predictor was simulated with an odds ratio (OR) per 1-SD of 2.0; the intercept was calibrated to reproduce each region’s observed prevalence of abnormal MRI. Reported values represent the proportion of simulations with $p<0.05$. These results indicate that regional analyses were adequately powered to detect large effects (AUC ≥0.75 or OR≈2.0), but underpowered for modest effects (AUC ≈0.65). Non-significant results in regional subgroup models should therefore be interpreted with caution as they may reflect sample size limitations rather than true absence of association.
